# Meeting the Burden of Self-management: Qualitative Study Investigating the Empowering Behaviors of Patients and Informal Caregivers

**DOI:** 10.2196/39174

**Published:** 2022-11-16

**Authors:** Therese Scott Duncan, Jon Engström, Sara Riggare, Maria Hägglund, Sabine Koch

**Affiliations:** 1 Health Informatics Centre Department of Learning, Informatics, Management and Ethics Karolinska Institutet Stockholm Sweden; 2 Stockholm Business School Stockholm University Stockholm Sweden; 3 Department of Women's and Children's Health Uppsala University Uppsala Sweden

**Keywords:** behaviors, chronic conditions, model of illness-related work, empowerment, self-management

## Abstract

**Background:**

Patient empowerment is an important concept and a movement toward person-centered care of patients with chronic conditions. Nevertheless, to date, most research on empowered patients or informal caregivers has been conducted from a narrow clinical perspective. Such research has mainly focused on how health care professionals can empower patients to increase self-care or compliance with treatment. Research on empowered patient and informal caregiver needs and self-empowering activities is scarce.

**Objective:**

We aimed to explore empowering behaviors from a patient and informal caregiver perspective in the context of self-management and to understand how health care can support such behaviors better.

**Methods:**

We used an exploratory, qualitative study design. A total of 15 semistructured interviews and 6 focus group interviews were conducted with 48 patients and informal caregivers. We analyzed the interviews using thematic analysis and used a directed content analysis to analyze the focus group interviews.

**Results:**

A total of 14 patterns of empowering behaviors were identified that were characterized by several exploratory and influencing activities performed by the participants. The participants expressed a desire to be more active in their care than what is expected and supported by health care professionals. The participants also desired better support for activities imposed on them by health care professionals.

**Conclusions:**

To enable a transformation of the health care system to better support self-empowering behaviors, there is a need to develop self-management approaches from a patient and informal caregiver perspective.

## Introduction

### Background

The academic discourse increasingly maintains the view of the patient as an empowered and knowledgeable participant leading the way for their peers in a difficult health care setting. The notion of the empowered patient is further conceptualized in different concepts in the literature. Ferguson and Frydman [1] wrote about the concept *e-patient* in the early 2000s, inspired by the digital development within society that was reflected in many patient behaviors [1]. e-Patient describes patients or informal caregivers (such as a family members or other persons with a close relationship to the patient) who use the internet to find health-related information and web-based peer communities. e-Patients are further described as “engaged, enabled, equipped and empowered” in relation to their health or in collaboration with health care professionals [2]. e-Patients have also been shown to generate their own health data which they learn from and share, as well as create innovative solutions from [3]. Furthermore, the Department of Health and Social Care in the United Kingdom introduced the concept of *expert patients*, to describe user-led self-management for chronic conditions [4]. Expert patients take the role of mentors to other patients, with their significant knowledge and skills in self-management and patient participation. Expert patients are recognized to be valuable in clinical situations, research, representing patients in committees, or lobbying to health care authorities [4,5]. Both concepts are related to the concept of *lead user* in the field of innovation research. Lead user theories describe individuals who create innovative solutions to meet their own specific needs and predict needs for the consumer population in general [6]. Notably, patient innovations can sometimes enable better coping strategies and improved self-management overall [7].

Although the general direction in research and practice increasingly acknowledges patients’ abilities and potential as meaningful partners in different domains of health care, we noted 2 important shortcomings in the literature [8-11]. The first shortcoming is that most research on patients’ or informal caregivers’ empowering behaviors has been conducted from the perspective of health care professionals. Such research primarily focuses on how patients and informal caregivers can contribute to health care and how they fit into the needs of the health care system [8-11], rather than what these individuals need from health care. According to Zimmerman [12], patient empowerment consists of 3 components: the belief in one’s own capability to influence the situation (intrapersonal component), the understanding of which actions to take to achieve a desired outcome (interactional component) and engaging in specific types of behaviors to exercise control and influence (behavioral component) [12]. However, Eskildsen et al [13] state that patients can only become empowered if given the opportunity by health care professionals [13]. Thereby, drawing upon the definition of empowerment as a relational concept [14] is dependent on health care professionals conveying power to a homogeneous group of patients [15]. This further emphasizes the health care professionals’ perspective. Studies on self-empowering aspects of patient empowerment are largely neglected in that perspective, and those aspects could be extended within the model of illness-related work describing patient self-management [16]. The second shortcoming we identified is that although the skills of empowered patients and informal caregivers have been described [2,17,18], there is still a lack of structured patterns of behaviors and the factors that influence them. This knowledge gap and the lack of a thorough examination of patients’ and informal caregivers’ needs, and expectations are addressed in this study. The aim of this study is to explore empowering behaviors from a patient and informal caregiver perspective in the context of self-management and to understand how health care can support such behaviors better.

### The Model of Illness-Related Work as Theoretical Background

The model of illness-related work by Corbin and Strauss [19] describes medical management, role management, and emotional management as tasks for gaining greater control when performing self-management [16,19]. The model lists 6 self-management skills: problem solving, decision-making, finding and utilizing resources, patient–health care professional collaboration, action planning, and adapting skills regarding one’s condition [9,20]. The model provides direction for nurses to practice and teach self-management strategies [16,19]. The model’s 3 self-management tasks provide a description of healthy and interventional behaviors. Medical management includes taking recommended medication, following directives for hygiene before, for example, surgery, or using assistive devices or tools to manage a disability. Role management describes how patients need to maintain or create new role-specific behaviors in line with their chronic condition. This can include navigating through the health care organization, finding new ways to perform physical activities, or finding correct information about their condition. Emotional management entails dealing with emotional aspects of having a lifelong condition, such as coping, depression, grief, and existential beliefs [16].

### The Taxonomy of Burden of Treatment as Theoretical Background

The 3 tasks presented in the model of illness-related work illustrate the complexity of managing a chronic condition. This complexity is further explained from a patient’s perspective by the taxonomy of burden of treatment [21]. To construct this taxonomy, patients with chronic conditions were asked to recount the structural burden they had to handle every day. These burdens included the need to coordinate between health care professionals, manage personal and economic factors owing to their condition, perform lifestyle changes, find information, and learn about their condition and create relationships (Multimedia Appendix 1 [1]). These burdens are described as being imposed on patients as they perform self-management and could lead to struggle with adherence to treatment and care, as well as poor quality of life [21,22].

## Methods

### Overview

This exploratory study followed a qualitative approach in 2 consecutive stages. The first stage consisted of semistructured interviews with 15 patients with chronic conditions or informal caregivers. All participants described themselves as highly empowered regarding their self-management and in collaboration with health care. The second stage consisted of 6 focus group interviews with a broader group of 33 patients with chronic conditions or informal caregivers. All participants were from different parts of Sweden. A total of 9 interviews were conducted via telephone and 6 via face-to-face interviews. All focus groups were performed physically in settings close to participants’ homes. The semistructured interviews were analyzed using thematic analysis and the resulting categories were used as key concepts to guide a directed content analysis of the focus group data.

### Recruitment and Sampling

Participants in both stages were recruited using purposive sampling [23,24]. For the semistructured interviews, participant recruitment was conducted across Sweden through a web-based announcement on a webpage from a project called “Patient Lead Users,” which addressed people with chronic conditions or their informal caregivers nationally. The announcement included a request for empowered patients and informal caregivers to nominate themselves or someone else as being actively engaged in collaboration with health care as well as self-management. Further inclusion criteria were age >18 years and having experienced ≥1 chronic condition. Of the 67 self-nominated or suggested participants, 10 (15%) patients and 5 (7%) informal caregivers were selected by the authors to cover different ways of being active within their self-management, as well as different chronic conditions, sex, age, and geographic locations. The study sample for stage 2 consisted of 33 participants distributed over 6 focus groups. In this stage, patients with chronic conditions and informal caregivers were approached and screened through patient associations or through employed peer support workers within different geographic regions in Sweden. This was done after analyzing stage 1. The inclusion criteria were aged > 18 years and had chronic conditions.

### Content Development

The semistructured interviews in stage 1 consisted of open-ended questions covering 4 themes: background, your health journey, health behaviors, and your role (Multimedia Appendix 2 [2]). The chosen themes were based on identified knowledge gaps in the literature; the lack of knowledge about patients’ and informal caregivers’ needs in relation to their health journey, how they act (health behaviors), and what influences their behaviors (your role). A total of 5 pilot interviews were conducted to test the questions provided in the interview guide. Data from the pilot interviews were not included in the study results. In stage 2, a multiple-category design was used with different types of participants and chronic conditions [25]. The questions in the protocol for the focus groups (semistructured interview guide, Multimedia Appendix 3 [3]) were developed from the analysis and results of the semistructured interviews conducted in stage 1.

### Data Collection

Data for both stages were collected by the first author and 4 coworkers from the Patient Lead User project between November 2017 and September 2019. The interviews consisted of 6 face-to-face interviews at a location convenient for the participants and 9 interviews over telephone when face-to-face interviews were not possible because of their condition or long distances. The interviews lasted an average of 44 minutes, with a total duration of 656 minutes, and a SD of 7.4. In stage 2, each focus group consisted of a moderator and 1 or 2 observers. The sessions lasted for an average of 103 minutes, with a total duration of 618 minutes, and a SD of 13.2. All focus groups were performed physically in settings close to participants’ homes. The semistructured interviews and focus group sessions were recorded and transcribed verbatim. Transcribed data were returned to those participants who requested it and provided feedback on the findings when necessary. Saturation was reached [24] after 12 interviews in the first stage and after 5 focus groups in the second stage. To verify the results, 3 additional semistructured interviews and 1 additional focus group were conducted. No further recruitment was necessary in addition to the original sample.

### Data Analysis

**Figure 1:**
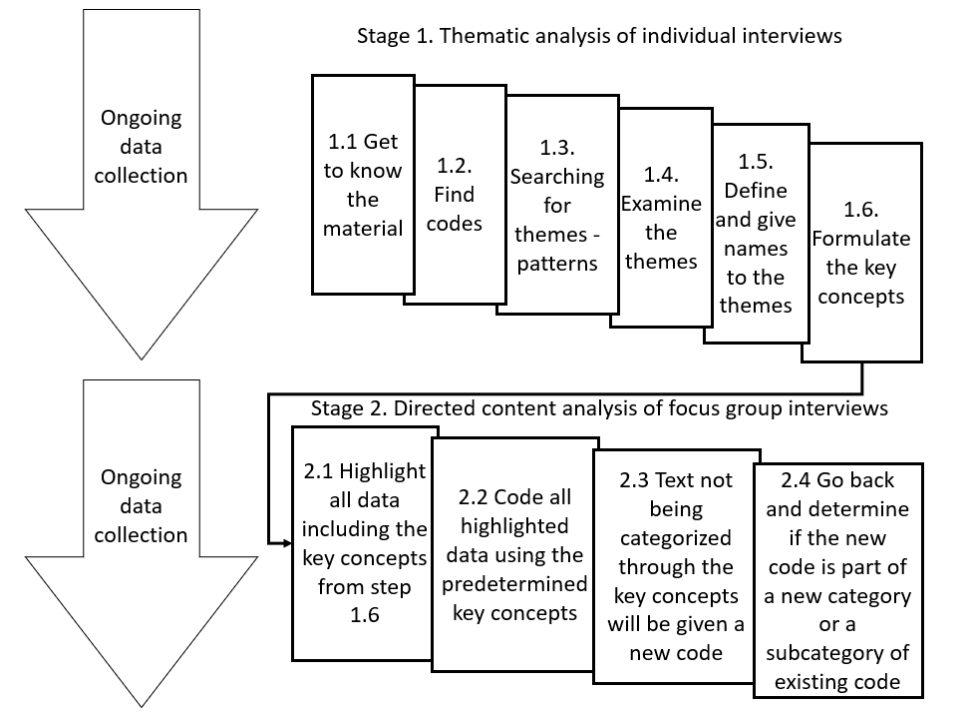
Illustration of the analysis for the whole study.

In stage 1, a thematic analysis was performed by all authors in parallel with the data collection. This is a flexible and inductive approach to analyze the data for the semistructured interviews [26,27]. Six phases were included: (1) familiarization to get acquainted with the data, (2) categorization of the data into units according to how the meaning of the data shifted, (3) finding patterns between the units to create themes, (4) situating all coded data into themes, (5) naming the themes according to their essence of how they fit into the aim of the study, and (6) formulating the key concepts from the categories within the themes [26]. The first stage resulted in 11 categories. These categories were used in stage 2 to further test and validate the knowledge from the semistructured interviews (Figure 1). Directed content analysis was used for data analysis [28,29], and the categories from stage 1 were used as key concepts to initiate the coding process. The authors followed 4 steps to examine how the categories emerged as behaviors related to self-management aspects [29]. (1) All data from the focus groups were coded and, when applicable, mapped into key concepts from the thematic analysis. (2) Subcategories were developed. (3) Data not matching one of the key concepts were assigned a new code, and (4) 3 new exploratory behaviors were established as categories.

### Ethics Approval

Ethics approval (decision 2015/1572-31/4 for interviews and 2018/2294-32 for focus groups) was provided by the Stockholm Regional Ethical Review Board. Written information about the purpose of the study, management of the data, and the option to opt out at any time was provided to the participants before the interviews and focus groups started. All participants signed informed consent forms after receiving oral and written information.

## Results

### Overview

**Figure 2:**
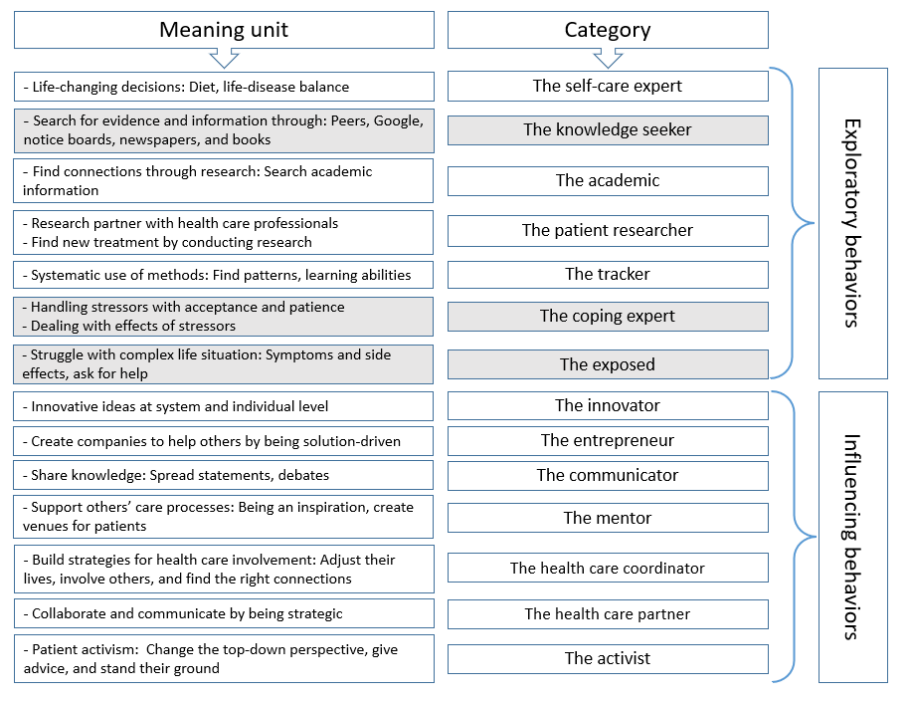
Illustration of key concepts and themes.

Participant characteristics included age, occupation during the time of interviews, years since diagnosis, sex, and different chronic conditions (Table 1 and Textbox 1). The analysis of the interviews resulted in 11 categories: *self-care expert, academic, patient researcher, tracker, innovator, entrepreneur, communicator, mentor, health care coordinator, health care partner,* and *activist* (see the white boxes in Figure 2). From the focus groups, 3 new exploratory behaviors were elicited: *knowledge seeker,* *coping expert,* and *exposed* (see gray boxes in Figure 2). The findings showed 2 major classes of empowering behaviors related to participants’ self-management activities: *exploratory* and *influencing behaviors*. These were illustrated as 2 overarching themes, where theme 1 described patterns of the exploratory phase of participants’ self-management, gaining experience, and knowledge of their condition. Theme 2 described patterns of the influencing part of self-management when the participants wanted to share their lived experience and knowledge with their peers and at the health care system level (Figure 2).

The participants adopted several patterns of behavior from both the themes, albeit often as a stepwise approach that spanned over several years and in different contexts. However, several of the exploratory patterns of behaviors were kept in parallel with the influencing patterns of behaviors, such as the self-care expert, knowledge seekers, academics, patient researchers, and trackers.

**Table 1:** Characteristics of participants (N=48).

Participant characteristic		Value, n (%)	
		Stage 1	Stage 2
**Age (years)**			
	18-45	5 (33)	8 (24)
	46-65	7 (47)	14 (43)
	>66	3 (20)	11 (33)
**Occupation**			
	Retired	4 (27)	15 (46)
	Sick leave	2 (13)	4 (12)
	Working	9 (60)	11 (33)
	Studying	N/A^a^	3 (9)
**Years since diagnosis**			
	<5	3 (20)	8 (24)
	6-10	7 (47)	13 (40)
	>10	5 (33)	12 (36)
**Sex**			
	Female	10 (67)	25 (76)
	Male	5 (33)	8 (24)
**Participant type**			
	Patient	10 (67)	27 (82)
	Informal caregiver	5 (33)	4 (12)
	Both	N/A	2 (6)

^a^N/A: not applicable.

**Chronic conditions presented by the participants.**

Chronic condition

- Brain neoplasms
- Breast neoplasms
- Colonic neoplasms
- Connective tissue disease
- Cyst-liver and Cyst-kidney
- Diabetes type 1 and 2
- Down syndrome
- Fatigue syndrome
- Fibromyalgia
- Heart condition
- Hypersensitivity
- Irritable bowel syndrome
- Kidney failure
- Kidney neoplasms
- Liver neoplasms
- Meningomyelocele
- Mental illness
- Motility disorder
- Multiple sclerosis
- Myalgic encephalomyelitis
- Myelodysplastic syndrome
- Myocardial infarction
- Ovarian neoplasms
- Parkinson disease
- Prostatic neoplasms
- Pulmonary fibroses
- Rheumatic disease
- Stroke
- Systemic sclerosis
- Thymus neoplasms
- Uterine neoplasms
- Whiplash injury

### Patterns of Exploratory Behaviors

Patterns of exploratory behaviors entail how participants contemplated their own situation and explored new ways to improve their situation. We identified the following patterns of behaviors: *self-care experts, knowledge seekers, academics, patient researchers, trackers, coping experts,* and *being exposed*.

By developing self-care strategies, making far-reaching lifestyle changes, and making life-changing decisions to create a balance in life, the *self-care expert* included patterns of behaviors found in several participants:

My clinician suggested dialysis for my kidney failure. However, I have Googled and knew it would give me 4-5 years to live, so I refused. I wished to get a transplant, if I still was alive after 5 years because of my kidney neoplasms. So, I asked my physician what I can do in my self-management to achieve that goal.

Patient from stage 1, male

Seeking knowledge from the literature, social media, or other digital sources is a major part of participants’ self-management. This provides for learning opportunities as a *knowledge seeker* or to perform a more systematic search of available literature, compiling research, finding connections to their own condition, and stay updated on the latest scientific articles and evidence as an *academic*. These proactive actions allow participants to find new ways of managing their condition and are often performed when the information from health care is insufficient:

The physician rarely talks about the future of my Parkinson disease. So, I push a bit because I have found information about something I want to test. Then I think about those who are not as well informed, do they not get the same care as I do?

Patient from stage 2, female

Suffering from two heart attacks, I decided it would not happen to me again. I started to read the literature of preventive measures, but it was too generic. So, I did my own review of scientific articles, to find the triggering aspect of my disease...

Patient from stage 1, male

Sometimes, an academic or knowledge seeker transforms into a *patient researcher* when developing a partnership with health care professionals to examine research to identify potential new treatment, use scientific methods to investigate their health issues, and engage as patient partners in research programs:

I found studies suggesting a biological medication for my systemic sclerosis. However, the medication was not yet approved. But with a great relationship and exchange concerning research, my physician helped by motivating a prescription for that medication...

Patient from stage 1, female

My therapist and I are “research friends.” Together we try to tackle new aspects of my mental illness... I think it is interesting since she does not try to be superior to me, even though she has a lot of knowledge... but we are on the same page regarding how to perform research together...

Patient from stage 2, female

The *tracker* includes patterns of behaviors found in several participants by systematically using methods to measure different health-related aspects (such as sleep, mood, food, physical activity, etc). The participants used digital technologies, paper and pen, or their mind to establish patterns, and learn through data to modify treatment or other health-promoting activities. This would be done to achieve deeper learning, better health, or better communication with health care professionals:

I adjusted time and dosage during the day, not exceeding my daily maximum dosage set by the physician. It resulted in me improving my health... and my self-efficacy regarding health care collaboration increased, since I realized that physicians can only give me guidelines regarding my Parkinson disease, then it is up to me to adjust according to my situation.

Patient from stage 1, female

Depending on how I feel and what feels relevant for me and my Multiple sclerosis, I perform self-tracking... It is related to food intake, and I have some classifications of how I measure health status. Then I also optimize my physical activity so I do not sit still all day and then believe I could compensate that with an hour at the gym. This is done with my smart watch, reminding me to move every hour...

Patient from stage 1, male

To emotionally cope with their self-management, some participants became *coping experts* dealing with stressors in their everyday life, either by changing their emotional responses to different stressors, which could include delaying difficult activities, or by dealing with the stressor itself. Restoring energy through physical activities and working with acceptance are some techniques they used to reduce existing problems:

I am driving motorcycle, traveling, and taking long hikes, until I am too sick. Because I know that the day will come when I need those mental pictures to be able to cope and trying to stay alive with my growing cyst-kidney and liver.

Patient from stage 2, female

I distanced myself from my Parkinson disease as long as possible... and I am quite happy since I am rather sensitive, so it was nice to be able to keep it all away from me. That I have not constantly thought of it for 11 years... instead I take it step by step. It has suited me well.

Patient from stage 2, female

Some participants also experienced being *exposed,* trying to collaborate with health care professionals and struggling with a complex life situation in society and within their family. They described an emotional struggle, sometimes hiding from society, feeling lonely, and not belonging anywhere. However, being exposed also included identifying problems that need to be solved:

The situation is complex since we [my husband and I] are both living with a chronic condition. I know how to live my life to manage my self-care for my Parkinson disease, but I’m not capable, since I need to take care of my husband with multiple diseases as well. But I cannot... leave him. I’m not there yet.

Patient and informal caregiver from stage 2, female

I worry when walking in the city, because of my whiplash injury. I can get very dizzy just stepping off a curb. And perhaps the police might think I’m drunk [laughing]…

Patient from stage 2, female

### Patterns of Influencing Behaviors on the Individual and System Level

The patterns of influencing behavior are those that may change the surrounding environment. Such patterns of behavior were exemplified as follows: *innovator, entrepreneur, communicator, mentor, health care coordinator, health care partner,* and *activist*. Good ideas based on health and health care needs were often based on previous lived experiences of chronic conditions and knowledge from their working life.

Exemplifying patterns of behaviors for *innovators* are the needs of performing self-care and collaborating with health care in a better way or to help in a community of peers based on experiences of lacking information related to their specific situation. Coming up with novel solutions for their situation was often accomplished by using digital or other physical solutions:

I met two other patients who have multiple sclerosis and that never had been in contact with someone they could relate to... Then the idea was born to try to reach out to other young patients by programming a webpage for this target group, since we felt it was empowering to be able to talk to someone who really understands you.

Patient from stage 1, male

Sometimes, innovators start organizations from their innovations, based on a strong need for more information or to help their peers. At other times, the participants become *entrepreneurs* based on their own health care experiences:

I was not given the correct treatment, which is very common for some diagnoses such as my genetic connective tissue disease, since there are no clear instructions for how to diagnose within primary care. The concept within the company is a process that has been digitalized and builds on trying to make it easier for primary care to refer you as a patient to the right specialist. If you do not get a referral to the right specialist, they do not know how to handle you.

Patient from stage 1, female

I started a publishing company and wrote and published several books about my healthcare experiences as an informal caregiver to my wife with pulmonary fibroses and liver neoplasms.

Informal caregiver from stage 1, male

Communicating with others about their lived experiences meant being a patient advocate, an inspiration, and making the disease visible. Several participants hoped to make a change for other peers in health care situations with their increased knowledge and acceptance in society, as well as using their professional knowledge. As a *communicator*, the participants could be working with companies, writing op-ed articles, using social media as a platform, or speaking at conferences in health care contexts:

Speaking at conferences or when writing article as an informal caregiver, there are two specific topics that I believe are connected: continuity and digitalization. To achieve a person-centered encounter, you need to combine that with the best suitable technology. I refuse to believe the choice is either to use technology or to have a physical encounter...

Informal caregiver from stage 1, female

I believe it is appreciated when I inform healthcare professionals at meetings and conferences, about my everyday life with Diabetes type 1... I believe it could lead to better treatments if we could collaborate in a new way... The patient has so much valuable information that is needed in health care as well, and that is where my heart is, trying to bridge a gap between health care and the patients.

Patient from stage 2, female

Whereas a *communicator* kept informing groups of people, others provide mentorship at an individual and personal level. By becoming role models for others through inspiration and paying it forward as a *mentor*, the participants made use of the knowledge they had acquired:

It is so rewarding to help my peers within Rheumatic disease... There are for example many people with foreign background in my region, and it is difficult for them to make themselves understood, and they might not ask for what they have the right to within society... I could work 100 percent just helping them, so they could improve their own chronic condition and health.

Patient from stage 1, female

I need to travel far for my treatment for Prostatic Neoplasms. Luckily there are three of us always traveling together, since we have convinced health care that we need to have slots suiting all three of us. This mean a lot, especially for one of us who is all alone with no family member supporting him. Now he could go with us and get support and not feeling alone in this...

Patient from stage 2, male

One major part of the participants’ self-management was coordinating their care at different health care sites. This required considerable knowledge, developing special skills to manage different actors around the patient, building relationships, and finding pathways to the right resources in a solution-oriented way of thinking as a *health care* *coordinator*. Often, the patient’s condition requires many health care contacts:

I need to coordinate primary care, the heart clinic and... the habilitation... as well as dental care since that is very important when having a heart failure. I have tried to make them all collaborate...

Informal caregiver from stage 1, female

Often, a deeper relationship and collaboration with health care professionals is crucial for the work as a health care coordinator. Some participants exemplified that their experience of a collaboration with health care professionals as *health care partners* is a great learning opportunity. Within these relationships, the participants also felt listened to and that health care professionals knew the participants had valuable information about their lives, for them to provide the best care. Even though increasing their collaboration with health care professionals was desirable, most of the participants experienced that it was difficult to achieve:

It has been challenging to represent my son in health care situations, since he is not good at explaining how he feels regarding his Down’s syndrome and heart failure... and to make health care professionals listen and to understand our situation. Before I would yell and scream. Now, I’ve learned it is more effective to lower your tone, to be taken seriously.

Informal caregiver from stage 1, female

I have been within health care with my rheumatic disease since I was 13 years old, and since it has been that many years an interest has grown within me to work with healthcare professionals and to become one myself.

Patient from stage 2, female

A few participants had the urge to change the health care system as they did not believe it was person-centered. They challenged the paternalistic structures trying to make a change in policies and structures related to their health care needs and health. These *activists* were acting as influencers on the web and offline. This was to help themselves and other peers stand their ground:

It is important to connect with people who are already interested in the topic, to be able to generate a change. There is no use banging one’s head against a wall... You can always start with a small change, and not wait for the structure to change. I believe it is important with this bottom-up-perspective.

Informal caregiver from stage 1, male

## Discussion

### Principal Findings

By exploring empowering behaviors in relation to self-management, we have identified different patterns that the participants follow. They are listed here with a descriptive term for each: *the self-care expert, the knowledge seeker, the academic, the patient researcher, the tracker, the coping expert, the exposed, the innovator, the entrepreneur, the communicator, the mentor, the health care coordinator, the health care partner,* and *the activist*. These patterns of behaviors are characterized by different activities that the participants perform for several years. None of the participants followed only one of these patterns but commonly adopted several patterns expanding over both exploratory and influencing behaviors. One example can be that a person starts with noticing a feeling of being exposed and vulnerable and addresses that by seeking knowledge and building self-management experience. Furthermore, the participants used that experience to make a difference for others and change the health care system through activism and new innovations. This empowering process is characterized by learning experiences and adapting to the current situation, depending on previous knowledge and existing skills. A common theme in the participants’ narratives, regardless of exploratory or influencing behaviors, was that they did not feel that they received support in their efforts from the health care system or society at large and sometimes even felt hindered when their behaviors were not in line with the expectations. The participants expressed having a desire to do much more than is currently expected by health care professionals, such as being a health care partner, an innovator, and a mentor. They also wanted better support for tasks that were not within their ability or interest but were imposed on them, such as acting as coordinators of their care.

With the current rather limited view on concepts such as self-management and patient empowerment within the existing literature and in the health care system, it is important to illustrate the concepts from a holistic perspective on patients and informal caregivers. This study contributes to the richness of empowering behaviors, illustrating how the participants extend their limits to influence the situation for themselves and for others. The participants know which actions to take within health care and in their self-care; however, the desired outcome is dependent on a functional collaboration with relevant actors. The study provides further evidence to the notion that patients and informal caregivers in some cases develop extraordinary behaviors and competencies that can serve as inspiration to others.

### Findings in Relation to Theoretical Background

Our results on empowering behaviors extend on the model of illness-related work [16]. By applying a patient and informal caregiver perspective, we uncovered additional categories of work that need to be undertaken in the case of illness. These categories of work can primarily be categorized as part of role management, entailing digital activities, working to improve the health care system, collaborating with health care professionals, and finding ways to navigate the health care system. Moreover, role management could also mean that the participants found new solutions for their needs, started companies to meet the needs of their peers, searched for information outside health care, became mentors spreading inspiration and information to their peers, tracked symptoms, and used proactive behaviors such as being self-care experts to prevent further disease by doing more than expected. To cope with everyday life and the feeling of being exposed are included in emotional management. However, we do see that most behaviors are part of role management, having to engage in activities that were not part of their life before the illness. Medical management was not explicitly included in our participants’ accounts, although we can see role management, such as the adjustment of medication based on tracking data being performed within the limits for medical management given by health care. This can be explained by the fact that the participants often had different views of what the most pressing tasks were rather than what was expected from the model of illness-related work. Emphasis on medical management was also a central theme in the patient empowerment literature, where the focus was on disease management and health care interaction [15]. Similarly, the concept of patient participation is most often narrowly described from a health care perspective [30]. One important contribution of this study was that, in contrast to the previous literature, our findings emphasized the self-empowering aspects of patient empowerment.

The taxonomy of the burden of treatment helped us describe the participants’ situation and understand where their behaviors arise from. The taxonomy of the burden of treatment describes how disease-related tasks are imposed on patients, how factors associated with these tasks are intensifying the burden, and how patients are affected by it [21]. In our results, the participants illustrated different situations of burden arising from personal circumstances and from the system level. The participants experienced everyday life burdens living with a chronic condition, such as dealing with stressors and feeling exposed within the society and family. Burdens could also entail a lack of collaboration with health care professionals, including insufficient information, not to be taken seriously, and being misdiagnosed. When it comes to coordinating health care at a system level, the participants illustrated challenging situations trying to navigate for themselves or for their next of kin within different health care situations. These burdens correspond well with the described aspects in the taxonomy [21], illustrating the burdens of lifestyle changes, nonworking collaboration with health care professionals, understanding of their condition and treatment, and emotional aspects. The empowering behaviors resulting from our participants’ narratives appeared as a paradoxical driving force toward increased autonomy and empowerment, moving forward from these obstacles in life. The participants understood that it was up to them to make changes within their self-care as health care professionals could only give them guidelines and not specific instructions to gain better well-being and health. By becoming mentors, communicators, and activists, the participants worked for change within the health care system. The participants shared what they learned through their lived experience and pursued a mutual learning experience with health care professionals.

### Strengths of Using Two Different Approaches of Data Collection

Our 2 different approaches to data collection gave us the opportunity to first gain an inductive and deep knowledge and thereafter to test the key concepts in 6 focus groups using an abductive approach. This also provided us with in-depth data when participants were inspired by each other and considered different aspects of their behaviors and activities than they would in single interviews. Including a larger group of participants mitigated the risk of self-management activities being performed only by powerful patients and informal caregivers with capital [31]. Self-management was performed by all participants; however, behaviors might differ depending on being an e-patient, expert patient, or lead user, or if the participant belonged to a late majority when it came to behavioral change. This was a process of reducing uncertainty regarding self-management behavior [32]. Behaviors performed by these early adopters are important for the development of self-management approaches, since the late majority of patients and informal caregivers tend to follow later on and make use of these solutions for better health [33].

### Limitations and Further Research

This study did not seek to perform a personality categorization of behaviors but instead illustrates different types of empowering behaviors as described by the participants. The sample was based solely on participants who were able to manage their or a family member’s condition. The consolidated criterion for reporting qualitative research—COREQ checklist—was used to ensure the reliability of the data and maintain transparency throughout the study (Multimedia Appendix 4) [34,35]. In addition, having empowering behaviors does not solve the power inequality in a paternalistic context [36]. Therefore, future research needs to consider the different types of behaviors from a health care perspective to explore how these behaviors are received by health care professionals and their rather limited understanding of patients’ self-management. This is important to increase the support from health care professionals.

### Conclusions

Keeping a strict patient and informal caregiver perspective, this study provides an in-depth understanding of the participants’ empowering behaviors and emphasizes the richness of self-empowering aspects of patient empowerment by extending on the model of illness-related work. This notion enables a perspective of what the participants can and want to do within their self-management and in collaboration with health care. The result illustrates how the participants extend their limits to influence the situation for themselves as well as for others in various ways and as a paradoxical driving force moving away from the obstacles illustrated by their everyday life stories, as well as described in the taxonomy of the burden of treatment. Today, patients and informal caregivers are part of a system that is not based on their needs; yet they are the main users. However, their behavior might differ depending on whether they are early adopters or late majorities when it comes to behavioral changes. Behaviors performed by early adopters are important for the development of self-management approaches as peers tend to follow later and make use of these solutions. To enable a transformation of the health care system to support patient empowerment and empowering behaviors, there is a need to develop solutions from a user perspective. This will increase the use of patient self-management and improve health care toward a more person-centric system.

## Appendix

Multimedia Appendix 1 The taxonomy of burden of treatment.

Multimedia Appendix 2 Interview guide: stage 1.

Multimedia Appendix 3 The protocol for focus groups: stage 2.

Multimedia Appendix 4 COREQ (consolidated criterion for reporting qualitative research) checklist.
