# Supplementary figures and images for: Meeting the Burden of Self-management: Qualitative Study Investigating the Empowering Behaviors of Patients and Informal Caregivers
Source: J Particip Med. 2022 Nov 16;14(1):e39174. doi: 10.2196/39174 (PMC9713617; doi:10.2196/39174)

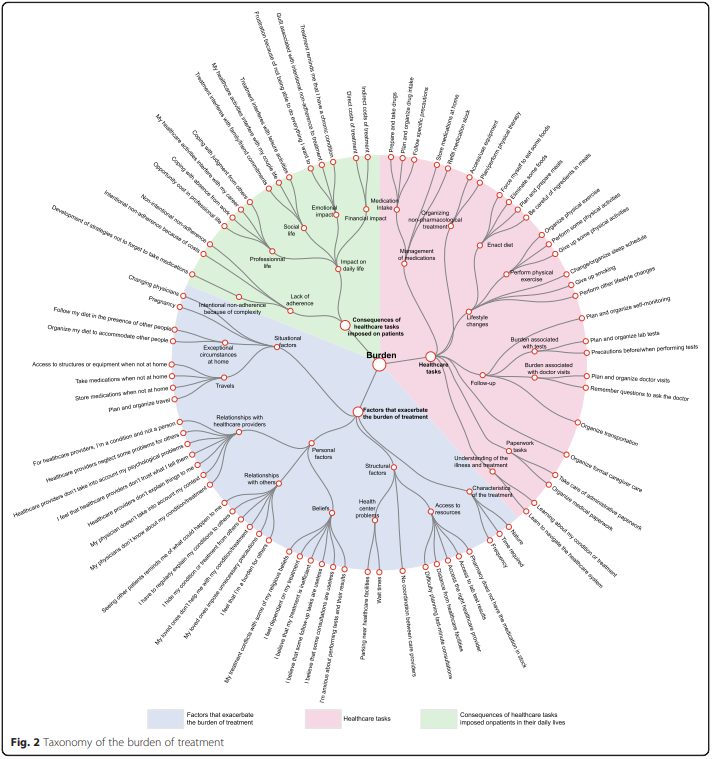

Supplement: Multimedia Appendix 1 [file jopm_v14i1e39174_app1.png]
