# Supplementary material for: Meeting the Burden of Self-management: Qualitative Study Investigating the Empowering Behaviors of Patients and Informal Caregivers
Source: J Particip Med. 2022 Nov 16;14(1):e39174. doi: 10.2196/39174 (PMC9713617; doi:10.2196/39174)
Supplement: Multimedia Appendix 2 [file jopm_v14i1e39174_app2.doc]

Multimedia Appendix 2 – Interview guide stage one

**BACKGROUND**

1. Please, briefly describe who you are.
2. Please, briefly describe your challenges regarding your health and disease.

MEDICAL HISTORY

1. Please, describe your medical history.

- Diagnosis and the principal symptoms.
- For how long?

**YOUR HEALTH JOURNEY**

1. Please, tell me about your situation.

- Please tell me about your life before the challenges you now are handling.
- What kind of needs are most important for you?

**HEALTH BEHAVIORS**

1. What do you believe you are doing, in comparison to others in the same situation?

- What kind of experience do you use?
- How do you get information?
- Which persons or other resources are important to you in your situation?
- What kind of tools do you use (e.g. technology)?

1. In what ways have you been able to affect your well-being?
2. What motivates you?
3. Do you interact with others in familiar situations?
4. Could you please tell me about a specific event that has been important to you, regarding your personal development?

**YOUR ROLE**

1. What is your opinion regarding your own role in relation to your health and well-being?

- How do you experience your role in relationship to healthcare?
- Have you got the support you need?
- Has any healthcare professional been more important to you, than others?
- What do you want healthcare to know about you?

1. How do you require the healthcare system to be?

**OTHER ISSUES**

1. Is there anything else you would like to talk about?
