# Supplementary material for: Meeting the Burden of Self-management: Qualitative Study Investigating the Empowering Behaviors of Patients and Informal Caregivers
Source: J Particip Med. 2022 Nov 16;14(1):e39174. doi: 10.2196/39174 (PMC9713617; doi:10.2196/39174)
Supplement: Multimedia Appendix 3 [file jopm_v14i1e39174_app3.doc]

Multimedia Appendix 3 – The protocol for the focus groups stage two

Before we start:

Do you have any questions about the study?

Is it okay to record the focus group?

Review the form of informed consent and sign it. Pointing out that participating is voluntary and they are free to interrupt the focus group at any time. They are also free to ask each other questions.

*Demographic:*

1. Can you please tell me your name, diagnosis (symptoms), time since diagnosis, age, and occupation?

*Everyday life activities regarding self-care or collaboration with health care:*

1. What kind of activities would you say you do because of your diagnosis?
2. Could you describe your journey from diagnosis to today?

- What have you learned?
- What kind of pre-knowledge and experience have you used?
- What kind of resources have you used?

1. In what ways has this affected you?

- What limitations have you experienced?

*Patterns of behaviors describing your activities:*

The moderator describes the 11 categories as symbols describing their activities/engagement. The participants are asked to move around in the room and mark the categories they feel connected to. They are also urged to think whether there is a category missing regarding their activities/engagement.

1. How did you experience this exercise?
2. Why do you believe this category is representing you?
3. Do these behaviors bring positive or negative feelings?
4. How can these behaviors change for you?

- Over time?
- Depending on different contexts?

1. What have we forgotten to discuss here today, that is important to you?
